# Dose-dependent effects of oral cannabidiol and delta-9-tetrahydrocannabinol on serum anandamide and related N-acylethanolamines in healthy volunteers

**DOI:** 10.1136/bmjment-2024-301027

**Published:** 2024-08-24

**Authors:** Timothy A Couttas, Carola Boost, Franziska Pahlisch, Eliska B Sykorova, Juliane K Mueller, Beverly Jieu, Judith E Leweke, Inga Dammann, Anna E Hoffmann, Martin Loeffler, Oliver Grimm, Frank Enning, Herta Flor, Andreas Meyer-Lindenberg, Dagmar Koethe, Cathrin Rohleder, F Markus Leweke

**Affiliations:** 1 Brain and Mind Centre, The University of Sydney, Camperdown, New South Wales, Australia; 2 Dept. of Psychiatry and Psychotherapy, Central Institute of Mental Health, Mannheim, Germany; 3 Endosane Pharmaceuticals GmbH, Berlin, Germany; 4 Dept. of Psychiatry, Psychosomatics and Psychotherapy, Goethe-Universitat Frankfurt am Main, Frankfurt am Main, Germany; 5 Dept. of Psychiatry and Psychotherapy, University of Goettingen, Goettingen, Germany; 6 Dept. of Cognitive and Clinical Neuroscience, Central Institute of Mental Health, Mannheim, Germany; 7 Dept. of Psychosomatic Medicine and Psychotherapy, Central Institute of Mental Health, Mannheim, Germany

**Keywords:** Schizophrenia & psychotic disorders, Data Interpretation, Statistical, PSYCHIATRY

## Abstract

**Background:**

The mental health benefits of cannabidiol (CBD) are promising but can be inconsistent, in part due to challenges in defining an individual’s effective dosage. In schizophrenia, alterations in anandamide (AEA) concentrations, an endocannabinoid (eCB) agonist of the eCB system, reflect positively on treatment with CBD. Here, we expanded this assessment to include eCBs alongside AEA congeners, comparing phytocannabinoids and dosage in a clinical setting.

**Methods:**

Liquid chromatography-tandem mass spectrometry quantified changes in serum levels of AEA, 2-arachidonoylglycerol (2-AG), alongside AEA-related compounds oleoylethanolamide (OEA) and palmitoylethanolamide (PEA), which were attained from two independent, parallel-designed, clinical trials investigating single, oral CBD (600 or 800 mg), delta-9-tetrahydrocannabinol (Δ^9^-THC, 10 or 20 mg) and combination administration (CBD|800 mg+Δ^9^-THC|20 mg) in healthy volunteers (HVs, n=75). Concentrations were measured at baseline (t=0), 65 and 160 min post administration.

**Results:**

CBD-led increases in AEA (1.6-fold), OEA and PEA (1.4-fold) were observed following a single 800 mg (p_corr_<0.05) but not 600 mg dosage. Declining AEA was observed with Δ^9^-THC at 10 mg (−1.3-fold) and 20 mg (−1.4-fold) but restored to baseline levels by 160 min. CBD+Δ^9^-THC yielded the highest increases in AEA (2.1-fold), OEA (1.9-fold) and PEA (1.8-fold) without reaching a maximal response.

**Conclusion:**

CBD-administered effects towards AEA, OEA and PEA are consistent with phase II trials reporting clinical improvement for acute schizophrenia (CBD≥800 mg). Including Δ^9^-THC appears to enhance the CBD-induced response towards AEA and its congeners. Our results warrant further investigations into the potential of these lipid-derived mediators as metabolic measures for CBD dose prescription and co-cannabinoid administration.

**WHAT IS ALREADY KNOWN ON THIS TOPIC**

- Cannabidiol (CBD) is purported to have therapeutic benefits for mental health conditions, with promising clinical evidence to support its medicinal value in the treatment of schizophrenia.
- Assigning a defined dose of CBD remains a challenge, where inconsistencies in efficacy could benefit from precise biological measures.

**WHAT THIS STUDY ADDS**

- In our investigation towards lipid-derived mediators of the endocannabinoid (eCB) system, we observed CBD positively impacted concentrations of anandamide (AEA) and its N-acylethanolamine (NAE) congeners.
- Endogenous effects were only observed at CBD dosages reported to have clinical benefit for acute schizophrenia (CBD≥800 mg).
- The inclusion of delta-9-tetrahydrocannabinol (Δ^9^-THC) seemingly heightened CBD’s endogenous effects.

**HOW THIS STUDY MIGHT AFFECT RESEARCH, PRACTICE OR POLICY**

- This study is the first to compare the effects of acute CBD administration alone and in a CBD-Δ^9^-THC combination on peripheral eCBs and NAEs.
- Our findings indicate that monitoring endogenous effects towards AEA and its congeners may provide theragnostic benefits for CBD applications.
- Our results support prior evidence of Δ^9^-THC interactions with CBD as an ‘entourage’ for increased benefit.

## Introduction

 Alterations to the endocannabinoid system (ECS) are implicated in the aetiology of neurological and psychiatric disorders, notably schizophrenia.^1^ The ECS consists of two endocannabinoids (eCBs), anandamide (AEA) and 2-arachidonoylglycerol (2-AG), which act as competing agonists for the binding of G-protein-coupled cannabinoid receptors (CB_1/2_-R) with delta-9-tetrahydrocannabinol (Δ^9^-THC), the main psychotomimetic component of *Cannabis sativa* (cannabis) that exacerbates schizophrenic psychoses.^2^,^4^ Influences on AEA have been strongly linked to Δ^9^-THC and cannabis consumption. Preclinical evidence has shown that repeated treatment of Δ^9^-THC downregulates AEA signalling in the rat striatum.^5^ In humans, concentrations of AEA were notably lower in the cerebrospinal fluid (CSF) of healthy volunteers (HV)^6^ and antipsychotic-naïve individuals with schizophrenia^7^ who were frequent cannabis users, compared with HVs and antipsychotic-naïve individuals with schizophrenia who were light cannabis users, respectively. Additionally, AEA exhibits protective properties early in the pathophysiology of schizophrenia.^8 9^

The principal non-psychotomimetic constituent of cannabis, cannabidiol (CBD), exerts clinical benefits either as a monotherapy or adjunctive treatment for schizophrenia,^10 11^ which are accompanied by a positive association with AEA.^10^ Though the mechanism is not conclusively understood, CBD’s low affinity for CB_1/2_-R has shifted research towards its capacity to modulate AEA degradation, alongside its congener N-acylethanolamines (NAEs), oleoylethanolamide (OEA) and palmitoylethanolamide (PEA).^12^ CBD’s optimal route of administration and appropriate dosage to achieve clinical efficacy remains a challenge, further complicated by the nature of the clinical syndrome(s) to be remedied.^13^ Hence, CBD’s clinical benefits may be underestimated due to an underappreciation of its biochemical activity. The study presented here is the first to explore effects on eCBs, alongside AEA congeners, following acute administration of CBD, Δ^9^-THC and combination therapy in phase I clinical trials, identifying measurable effects on these lipid mediators that appear dose dependent.

## Methods

Human serum (n=75) was collected from two of our previous phase I clinical trials, exploring clinical and fMRI responses to CBD and Δ^9^-THC administration in HVs (LOGIN-TS4, GermanCTR: DRKS00005442; GEI-TCP-II, ClinicalTrials.gov: NCT02487381).^14 15^ Participant demographics were relatively balanced between the respective groups (table 1).

**Table 1:** Demographics and eCB/NAE measurements prior to the commencement of analysis

	CBD|800 mg (n=13)	CBD|600 mg (n=10)	CBD|800 mg+Δ^9^-THC|20 mg (n=12)	THC|20 mg (n=12)	THC|10 mg (n=10)	Placebo (n=18)	Total (n=75)
Age	24.85 (1.21)	25.50 (1.55)	24.17 (1.00)	23.00 (0.82)	22.90 (1.08)	24.11 (0.89)	24.09 (0.44)
Weight (kg)	81.80 (2.93)*	76.50 (3.28)	77.08 (2.11)	76.28 (2.38)	75.70 (2.49)	76.09 (2.88)	77.31 (1.14)
Height (cm)	180.00 (1.56)	180.20 (1.61)	183.83 (1.94)	179.83 (2.69)	182.60 (1.98)	178.89 (2.07)	180.64 (0.85)
BMI (kg/cm^2^)	25.25 (0.84)	23.52 (0.84)	22.80 (0.49)	23.70 (0.94)	22.67 (0.47)	23.66 (0.61)	23.68 (0.31)
Smoker (no/yes)	8/5	9/1	10/2	10/2	9/1	12/6	58/17
Cigarettes per day	2.62 (1.58)	0.10 (0.10)	1.17 (1.00)	1.17 (0.87)	0.20 (0.20)	2.06 (1.25)	1.36 (0.46)
Cannabis lifetime use	5.38 (0.73)	0.70 (0.50)	7.17 (0.61)	4.67 (0.50)	1.30 (0.50)	3.67 (0.70)	3.97 (0.35)
Prescreen eCB/NAE concentrations (pmol/mL)†							
AEA	1.37 (0.21)	2.00 (0.26)	1.03 (0.08)*	1.24 (0.11)	2.34 (0.25)*	1.55 (0.10)	1.55 (0.08)
2-AG	2.46 (0.47)	4.49 (1.36)	2.16 (0.23)	3.45 (0.42)	3.69 (0.62)	2.86 (0.76)	3.10 (0.30)
OEA	7.18 (0.95)	7.05 (0.58)	6.14 (0.47)	10.54 (1.51)*	7.03 (0.62)	8.07 (0.86)	7.73 (0.40)
PEA	18.22 (2.46)	20.68 (2.84)	16.30 (1.26)	21.30 (1.71)	19.78 (1.80)	12.02 (1.25)*	17.26 (0.84)

* A source of variation when comparing cohorts for a particular demographic/eCB/NAE (two-way analysis of variance, p<0.05, Benjamini, Krieger and Yekutieli method) to the overall (n=75) average.

† All values (when available) are reported as the mean±SE.

AEA, anandamide; 2-AG, 2-arachidonoylglycerol; BMI, body mass index; CBD, cannabidiol; eCB, endocannabinoid; NAE, N-acylethanolamine; OEA, oleoylethanolamide; PEA, palmitoylethanolamide; Δ9-THC, delta-9-tetrahydrocannabinol.

**Figure 1:**
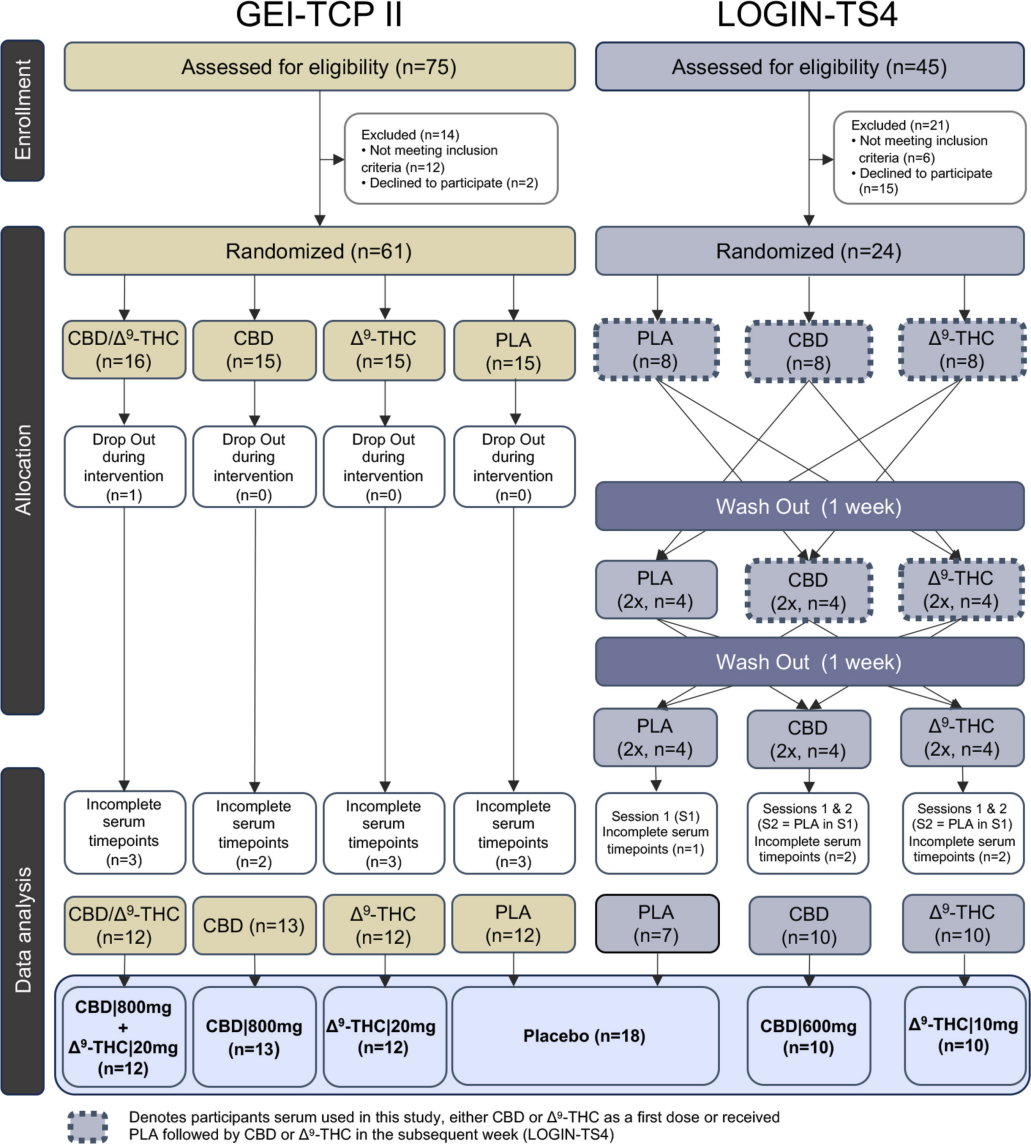
Consolidated Standards of Reporting Trials diagram of study designs and participant’s serum collection from respective phase I clinical trials (GEI-TCP-II and LOGIN-TS4). CBD, cannabidiol; Δ^9^-THC, delta-9-tetrahydrocannabinol; PLA, placebo.

Eligible participants included male adults aged 18–45 years with a body mass index between 18 and 30 kg/m^2^. Participants in their respective clinical trials received the same vegetarian meals provided by the Central Institute of Mental Health hospital kitchen. GEI-TCP-II participants (n=61) received a single dose of placebo (n=15), Δ^9^-THC (20 mg, n=15), CBD (800 mg, n=15) or combination (CBD|800 mg+Δ^9^-THC|20 mg, n=16) in a double-dummy design. A single participant dropped out during the intervention (CBD|800 mg+Δ^9^-THC|20 mg), and 10 participants did not complete serum collections at time points that corresponded with the LOGIN-TS4 study (figure 1), hence were omitted from our analysis.

LOGIN-TS4 HVs (n=24) were part of a three-arm, cross-over, single-dose administration of placebo, Δ^9^-THC (10 mg) and CBD (600 mg). The order of drugs was randomised between participants, ensuring equal numbers (n=8) of drug administration before each series of measurements, with a 1-week washout period between subsequent doses (figure 1). For our study, only serum collected in weeks 1 and 2 for CBD/Δ^9^-THC recipients who received the placebo in week 1 was assessed. This avoided the risk of residual phytocannabinoids and potential discrepancies in eCBs/NAEs concentrations. This resulted in a total of 8 times placebo, 12 times CBD (n=4 after placebo in week 1) and 12 times Δ^9^-THC (n=4 after placebo in week 1) HVs for each group (figure 1). Six participants were excluded (1 time placebo, 3 times CBD, 2 times Δ^9^-THC) who did not complete the time collection points that mirrored GEI-TCP-II.

The eCBs, NAEs and CBD/Δ^9^-THC (if received) were extracted from 1 mL serum following the addition of internal standards as previously described.^16^ Blood draws were collected and assessed in parallel time points for each study: baseline (t=0), 65 and 160 min post administration. Bloods were also taken at a pre-screening (PS) visit the week prior. Liquid Chromatography-Tandem Mass Spectrometry (LC-MS/MS) was performed on a TSQ Altis coupled to a Vanquish HPLC system (ThermoFisher), with analytes separated on a 4 µm Synergi Hydro-RP C18 column (150×2 mm; Phenomenex, Torrance, California, USA) over an 18 min gradient, as previous.^16^ Operational parameters for the specific eCBs, NAEs, CBD and Δ^9^-THC transitions monitored are listed in online supplemental table 1.

Peak integration and quantification were performed using Xcalibur (ThermoFisher), with peak areas normalised to their respective deuterated internal standards. All analytes quantified were expressed as pmol/mL. Endogenous effects to eCBs and NAEs following acute drug administration were analysed by two-way analysis of variance (repeated measures), corrected for multiple comparisons using the Benjamini-Krieger-Yekutieli post-test (GraphPad Prism, V.9.1.0). For all experiments, significant changes in eCBs and NAEs from baseline concentrations (t=0) were established at p_corr_<0.05.

Correlations (r) on statistically significant changes to eCBs/NAEs with phytocannabinoids were performed using Spearman analyses. Phytocannabinoid concentrations were compared against serum AEA/NAEs changes, with 65 and 160 min concentrations subtracted from baseline t=0 (Δpmol/mL). To assess for concomitant changes with fatty acid amide hydrolase (FAAH)-selective analytes, associations between AEA with OEA and PEA were evaluated, both as concentrations that incorporated all time-points, including pre-screen, as well as Δpmol/mL at 65 and 160 min, with p values less than 0.05 considered significant. Outliers (ROUT method, Q=1%, GraphPad Prism, V.9.1.0) were removed prior to all correlations assessed.

## Results

### Baseline characteristics and CBD/Δ^9^-THC exposure

**Figure 2:**
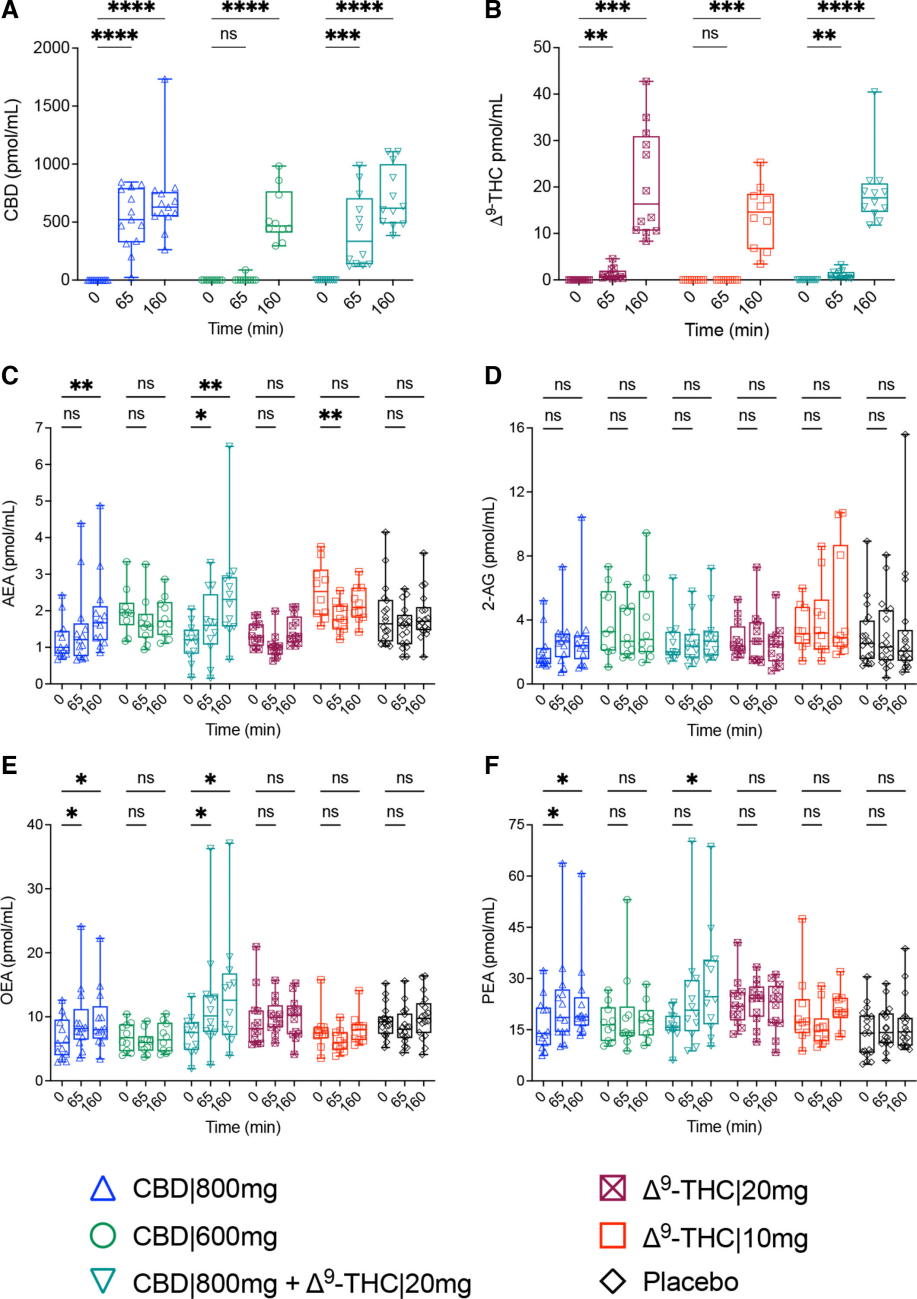
Measured endocannabinoids and N-acylethanolamines in human serum following acute administration with CBD|800 mg (blue triangle), CBD|600 mg (green circle), Δ^9^-THC|20 mg (maroon crossed-square), Δ^9^-THC|10 mg (red square), in-combination (CBD|800 mg+Δ^9^-THC|20 mg, teal triangle) and placebo (black diamond). Box and whisker plots display concentrations (pmol/mL) for (A) CBD, (B) Δ^9^-THC, (C) AEA, (D) 2-AG, (E) OEA and (F) PEA at baseline (t=0), as well as 65 and 160 min post dose. Statistical significance was determined by two-way repeated measures analysis of variance, which were adjusted for multiple comparisons using Benjamini-Krieger-Yekutieli false discovery rate (*p_corr_<0.05, **p_corr_<0.01, ***p_corr_<0.001, ****p_corr_<0.0001 represent significant changes at 65 and 160 min, compared with t=0; ns, not significant). AEA, anandamide; 2-AG, 2-arachidonoylglycerol; CBD, cannabidiol; OEA, oleoylethanolamide; PEA, palmitoylethanolamide; Δ9-THC, delta-9-tetrahydrocannabinol.

Variabilities in analyte concentrations, comparing each respective treatment group average (mean±SE, pmol/mL) to the combination of all participants, were observed for AEA (CBD|800 mg+Δ^9^-THC|20 mg, Δ^9^-THC|10 mg), OEA (Δ^9^-THC|20 mg) and PEA (placebo) during our PS assessments, (table 1), as well as for AEA (Δ^9^-THC|10 mg) at t=0 (online supplemental figure 1). However, these analytes were deemed stable at the commencement of the study as their concentrations aligned with those previously reported^17 18^ and remained analogous between PS and t=0 measurements, displaying no significant alterations between time-points prior to intervention (online supplemental figure 1). For all HVs, serum levels of exogenous CBD and Δ^9^-THC were sufficient by 160 min to expect physiological effects,^19^ with CBD and Δ^9^-THC administered at higher doses (800 vs 600 mg and 20 vs 10 mg, respectively) displaying a faster rate of accrual (figure 2A,B).

### AEA and congener changes in response to phytocannabinoid dosage and combinations

An initial effect towards AEA was observed on administration of Δ^9^-THC, leading to a decrease in concentration at 65 min (Δ^9^-THC|10 mg, −1.4-fold, p_corr_=0.0014; THC|20 mg, −1.3-fold, p_corr_=0.1160). However, by 165 min, levels had returned to t=0 levels (figure 2C). In contrast, CBD administered at 800 mg demonstrated a continued increase in AEA concentration (65 min, 1.3-fold, p_corr_=0.0514; 160 min, 1.6-fold p_corr_=0.0030), with the combination treatment (CBD|800mg+Δ^9^-THC|20 mg) inducing an even greater response (65 min, 1.4-fold, p_corr_=0.0328; 160 min, 2.1-fold, p_corr_=0.0080) (figure 2C). No reported differences in AEA concentrations were observed with CBD|600 mg (figure 2C). Neither CBD nor Δ^9^-THC significantly influenced 2-AG at any time or dosage (figure 2D).

OEA and PEA concentrations increased, in concert with their structural analogue AEA, following the intake of CBD|800 mg (65 min: OEA, 1.4-fold, p_corr_=0.0132; PEA, 1.4-fold, p_corr_=0.0478) and CBD|800mg+Δ^9^-THC|20 mg (65 min: OEA, 1.7-fold, p_corr_=0.0303; PEA, 1.5-fold p_corr_=0.0520). CBD|800 mg mediated changes appeared to have reached their maximal response (165 min: OEA, 1.4-fold p_corr_=0.0132; PEA, 1.4-fold p_corr_=0.0405) while effects following CBD|800mg+Δ^9^-THC|20 mg (OEA: 1.9-fold, p_corr_=0.0234; PEA, 1.8-fold p_corr_=0.0190) continued over the course of the analysis (figure 2E,F). The mean concentrations (pmol/mL) for eCBs and NAEs at each time-point and treatment group are provided alongside the individual and adjusted p values (p_corr_) in online supplemental table 2.

**Figure 3:**
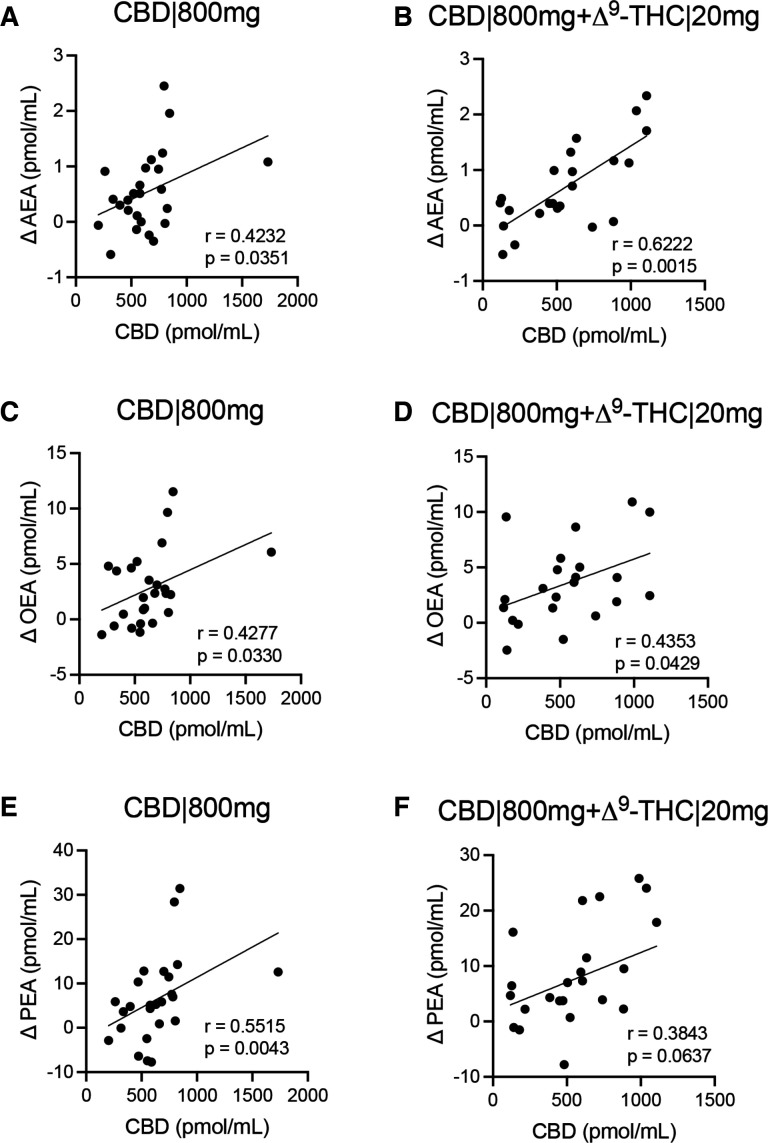
Associations of CBD with changes to baseline levels (t=0) of (A,B) AEA, (C,D) OEA and (E,F) PEA. The differences at 65 and 165 min are presented on the y-axis (Δ pmol/mL) against CBD concentrations for same time-points on the x-axis (pmol/mL) for (A,C,E) CBD|800 mg and (B,D,F) CBD|800mg+Δ^9^-THC|20 mg. Correlations were determined by Spearman analysis at a CI of 95%. The coefficient of correlation (r) and p values are shown. AEA, anandamide; CBD, cannabidiol; OEA, oleoylethanolamide; PEA, palmitoylethanolamide; Δ9-THC, delta-9-tetrahydrocannabinol.

Spearman analyses confirmed that increasing concentrations of CBD at 65 and 160 min positively associated with changes (Δpmol/mL) in AEA (CBD|800 mg, r=0.4232, p=0.0351; CBD|800mg+Δ^9^-THC|20 mg, r=0.6222, p=0.0015), OEA (CBD|800 mg, r=0.4277, p=0.0330; CBD|800mg+Δ^9^-THC|20 mg, r=0.4353, p=0.0429) and PEA (CBD|800 mg, r=0.5515, p=0.0043; CBD|800mg+Δ^9^-THC|20 mg, r=0.3843, p=0.0637), when administered at 800 mg CBD, with or without the addition of Δ^9^-THC (figure 3). Positive associations between AEA and NAEs were also consistent across participants in both CBD|800 mg and CBD|800mg+THC|20 mg treatment arms, both as change from t=0 (Δpmol/mL) and when comparing concentrations across all time-points measured, including PS values (pmol/mL) for all participants (online supplemental figure 2). Concentrations of Δ^9^-THC|10 mg were below the limit of detection to yield values for association with AEA at 65 min (data not shown). However, we did demonstrate a negative association for Δ^9^-THC|20 mg with AEA (r=−0.4098, p=0.1859), with OEA and PEA not displaying any directed association towards Δ^9^-THC|20 mg (r<0.1, online supplemental figure 3A,C,E). In contrast, Δ^9^-THC levels were positively associated with AEA, OEA and PEA when coadministered with CBD|800 mg (online supplemental figure 3D,E,F).

## Discussion

This study sought to observe relative changes to eCBs and NAEs in the serum of HVs following oral phytocannabinoid administration in a retrospective investigation that measured matched time-points from two completed clinical trials (LOGIN-TS4, GEI-TCP-II). We postulate that the observed changes for CBD|800 mg (AEA, OEA and PEA), Δ^9^-THC (AEA) and in-combination (CBD|800mg+Δ^9^-THC|20 mg; AEA, OEA and PEA) represent mechanistically relevant responses, given no significant influences in their endogenous levels were detected prior to phytocannabinoid administration.

Increased levels of AEA from CBD|800 mg were consistent with our prior findings for CBD treatment of schizophrenia, leading to increased sera AEA associated with improved clinical symptoms.^10^ Reports suggest CBD-induced increase is caused by inhibiting FAAH, AEA’s principal metabolising enzyme, verified in rat brain membranes,^10^ mouse brain microsomes^12^ and COS-7 cells expressing rodent FAAH.^12^ However, CBD has not been reported to inhibit FAAH in humans, rather blocking fatty acid binding proteins (FABPs) intracellular transport of AEA to FAAH at the endoplasmic reticulum.^12^ Although the relevance of this mechanism remains conjectural, CBD’s capacity to block FABPs-mediated transport to FAAH would also lead to increases in OEA and PEA, given these endogenous lipid mediators share this conserved hydrolysis pathway with AEA.^12^ Significant increases in OEA and PEA were observed following CBD administration, aligning with the proposed FABPs–FAAH mechanism of inhibition (figure 2E,F).

Though evidently more complex, it is widely acknowledged that NAEs follow a canonical biosynthesis pathway initiated by the N-acylation of membrane glycerophospholipids.^20^,^22^ This process results in the formation of N-acyl-phosphatidylethanolamine (NAPE) precursors, which undergoes specific phospholipase D type (NAPE-PLD) hydrolysis, with N-arachidonoyl-phosphatidylethanolamine, N-oleoyl-phosphatidylethanolamine and N-palmitoyl-phosphatidylethanolamine each being converted into AEA, OEA and PEA, respectively.^23 24^ Given that NAPE-PLD is integral to the NAE metabolic process, an alternative, although speculative, viewpoint is that CBD induces concomitant increases in AEA and its congeners through increased biosynthesis. Indeed, CBD has been shown to promote the upregulation of NAEs in multiple brain regions of rats via an NAPE-PLD-dependent mechanism,^25^ as well as enhance mRNA expression of NAPE-PLD in syncytiotrophoblasts.^26^

Increases in AEA, OEA and PEA occurred only from the administration of CBD at 800 mg, alone or in combination with Δ^9^-THC. This metabolic response aligns with the dose-dependent clinical efficacy of CBD. In our previous report, 800 mg of CBD significantly improved schizophrenia symptoms,^10^ while clinical trials reported CBD at 600 mg, as an add-on therapy, was insufficient to improve cognitive function or attenuate psychotic symptoms,^27^ though the latter study did observe increased connectivity during resting state.^28^ In support of a ≥800 mg threshold, CBD as an add-on to conventional antipsychotics does exhibit clinical benefit at 1000 mg,^11^ while displaying no positive effects when given as a CBD/Δ^9^-THC mix (Bedrolite, with CBD ca. 300 mg/day) to patients with a psychotic disorder and comorbid cannabis use.^29^

CBD’s poor oral bioavailability (estimated at 4%–6%^30^) could explain why benefits and induced effects on AEA/NAEs require the high dosage, as 800 mg CBD exhibits a faster accrual, over 600 mg (figure 2A). This same rationale may also account for comparable effects towards AEA following Δ^9^-THC at different doses, where their accumulation rates and subsequent impact on AEA were similar (figure 2B). Analogous declines in AEA concentrations of the CSF have been reported in studies comparing heavy (>10 times/month) to light (<10 times/month) cannabis consumers without any diagnosis of psychopathology according to Diagnostic and Statistical Manual of Mental Disorders, version IV (DSM-IV) criteria,^6^ as well as in individuals diagnosed with schizophrenia and higher consumption of cannabis in their lifetime (>20 times/life) compared with those with no or sporadic use (<5 occasions in their lifetime).^7^ However, it should be noted that in the latter study, a subgroup of high-frequency cannabis users with schizophrenia tested positive for urinary Δ^9^-THC still displayed elevated AEA in the CSF, though statistical significance was not reached. Therefore, the overall lower AEA levels in high-frequency cannabis users diagnosed with schizophrenia may not be attributed to recent cannabis use.

Previous reports on alterations in circulating AEA due to Δ^9^-THC and cannabis consumption have shown considerable variability. The aforementioned studies found no effects on human sera,^6 7^ while others reported increases in AEA alongside mixed effects on OEA and PEA levels in participants undertaking^31^ or undergoing cessation^32^ from high-cannabis use. Lower AEA concentrations have been observed in the plasma of individuals with psychosis who self-reported high-cannabis use,^33^ whereas higher AEA levels were found in individuals with cannabis use disorder, accompanied by concomitant increases in OEA.^34^ Clinical evidence supports elevated eCB/NAE response following acute, oral administration of Δ^9^-THC at a dosage identical (20 mg) to a treatment arm used in this study, with results displaying higher levels of AEA alongside 2-AG and OEA in plasma 2–3 hours post in-take.^35^ Conversely, a longer clinical trial reported a decline in plasma AEA over 28 days in participants undergoing cessation for their cannabis use disorder, with stable AEA levels in participants that received 800 mg of CBD, while this effect was absent in those receiving 400 mg of CBD.^36^ The contradictory reports of AEA/NAE levels may be a consequence of their biphasic response towards Δ^9^-THC, which is postulated to be mediated by synthesis and/or degradation processes^37 38^ or through catecholaminergic and glucocorticoid signalling that, in turn, promote eCB/NAE concentrations.^37^,^41^ Thieme *et al* reported that plasma concentrations of AEA and 2-AG undergo repeated changes (both upregulation and downregulation) at multiple time points up to 48 hours post intravenous injections of Δ^9^-THC.^38^ This infers that those acute investigations, including this study, may have limitations in interpreting the effects of Δ^9^-THC on AEA and its congeners. Additionally, formulation and mode of administration could also impact the response to Δ^9^-THC^16 42^ and its combination with CBD,^37^ as discussed below.

OEA and PEA concentrations appeared to plateau with CBD-alone treatment, suggesting that the maximal affinity for inhibiting their degradation may have been reached. This is postulated to be due to either a higher affinity of FAAH for AEA^43^ or the metabolism of OEA and PEA via alternative pathways, such as N-acylethanolamine acid amidase (NAAA),^44^ which is not primarily responsible for the hydrolytic deactivation of AEA,^45^ nor inhibited by CBD.^46^ Additionally, NAPE-PLD is not exclusively responsible for the formation of NAEs.^47^ Alternative pathways involve phospholipase C, phospholipase A or α/β Hydrolase Domain-Containing Protein 4 catalytic hydrolysis, as reviewed by Simard *et al*.^48^ While the importance of these multiple pathways remains unknown, they may facilitate the differential synthesis of AEA, OEA and PEA, potentially varying based on the specific tissue or physiological process involved.^49^ Differential effects have also been noted across the overall pathway, with preclinical models recognising FAAH as a critical step for regulating NAEs in the brain but not heart tissue.^50^ Hence, identifying the source(s) of our circulatory AEA/NAE alterations could elucidate relevant aspects or overlapping mechanisms of the metabolic pathway contributing to the dose-dependent changes observed with CBD and Δ^9^-THC. However, resolving the origin of circulating eCBs/NAEs presents a challenge, owing to their lipophilic nature, which means they are identifiable in almost every tissue and produced by nearly all cell types.^51^

In this study, NAEs continued to increase, alongside a greater upturn of AEA, from the combination of CBD+Δ^9^-THC, leading us to speculate a positive contribution towards CBD-derived mechanisms occurs with the inclusion of Δ^9^-THC (‘entourage effect’).^52^ Though beyond this project’s scope, prior evidence of CBD+Δ^9^-THC therapy supports analgesic efficacy^53^; however, safety and tolerance of Δ^9^-THC remain debated.^54^ Furthermore, single-dose administration of CBD+Δ^9^-THC through inhalation yielded no effect on eCB/NAE levels.^37^ It is important to note that doses administered by Chester *et al* were designed to reflect recreational cannabis use, with CBD doses of only up to 30 mg. Additionally, comparisons between formulations are challenging due to the delayed absorption and metabolism from the first-pass effect of oral phytocannabinoid administration.^55^ Refinement of Δ^9^-THC dosage and pharmacokinetics to support CBD efficacy for certain indications while minimising adverse effects warrants future investigation.

Our study has several limitations. Though endogenous effects were observed, our sample size remains relatively small. This may account for eCB/NAE changes higher in dosage (e.g., Δ^9^-THC 20|mg effects on AEA) or magnitude (CBD|800 mg+Δ^9^-THC|20 mg response on PEA at 65 min) did not reach statistical significance. The exploratory nature of combining randomised clinical trials, along with the increased likelihood of variation in results when working with small sample sizes, constrains our comparative measures to within-treatment arms. This underscores the necessity of addressing these limitations in expanded studies to authenticate our observations. In particular, clinical trials that allow for more controllable comparisons between CBD and Δ^9^-THC (including combined effects), increased sampling times that better encapsulate endogenous alterations or validate non-apparent responses observed herein (eg, CBD|600 mg) and, if permissible, higher concentrations or longer administrations to clarify whether endogenous effects are dose-dependent or reflect a pre-existing state. Influences on endogenous eCBs/NAEs, and the required dosage to achieve them, may also have been affected by food intake. Phytocannabinoid bioavailability increases with high-fat meals, likely due to their lipophilicity requiring intestinal lumen solubilisation before absorption.^56^ Although participants in both studies received standardised meals, they were not identical. This may have slightly belated the absorption of CBD and Δ^9^-THC between the studies (GEI-TCP-II and LOGIN-TS4), delaying any subsequent effects on AEA and NAEs. However, this would not account for differences between CBD-alone versus combination (CBD+Δ^9^-THC), which were controlled in the same study and administered with an identical dosage of CBD. Finally, we also acknowledge that this study does not address the question of whether the observed peripheral changes are indicative of a cerebral response. Although our observations are consistent with the reported clinical benefits of CBD^10 11^ and Δ^9^-THC effects on the CSF,^6 7^ response in peripheral systems may differ from cerebral effects, as there is evidence of a lack of association between these two biofluids.^57 58^ To the best of our knowledge, no comparative investigations have yet to assess CBD’s effects on the eCBs and NAEs in the CSF alongside paired peripheral fluids.

In summary, our data supports both previous clinical and mechanistic evidence for AEA’s and NAEs’ response to phytocannabinoids. Our results could reflect a dose-dependent metabolic signature for CBD, with potential enhancement from Δ^9^-THC, encouraging future investigations into these endogenous lipid mediators as indicative markers of CBD efficacy, both as a stand-alone or co-administered cannabinoid therapy.

## Supplementary material

10.1136/bmjment-2024-301027 online supplemental figure 1

10.1136/bmjment-2024-301027 online supplemental figure 2

10.1136/bmjment-2024-301027 online supplemental figure 3

10.1136/bmjment-2024-301027 online supplemental table 1

10.1136/bmjment-2024-301027 online supplemental table 2

## Data Availability

All data relevant to the study are included in the article or uploaded as online supplemental information.
