# Supplementary material for: Dose-dependent effects of oral cannabidiol and delta-9-tetrahydrocannabinol on serum anandamide and related N-acylethanolamines in healthy volunteers
Source: BMJ Ment Health. 2024 Aug 24;27(1):e301027. doi: 10.1136/bmjment-2024-301027 (PMC11409355; doi:10.1136/bmjment-2024-301027)
Supplement: online supplemental figure 1 [file bmjment-27-1-s001.pdf]

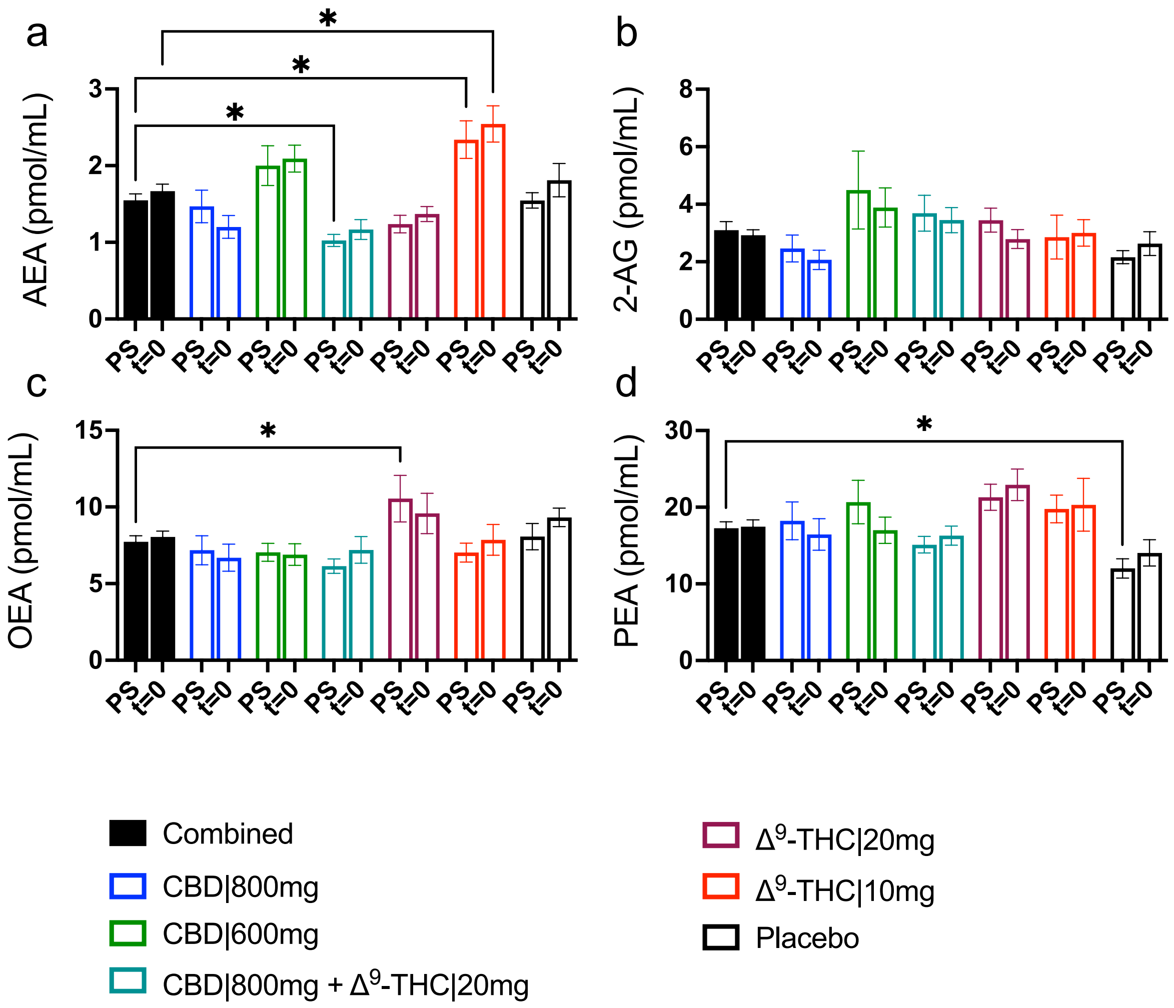

Supplementary Figure 1. Endogenous (A) AEA, (B) 2-AG, (C) OEA and (D) PEA concentrations at pre-screen (PS) and baseline (t=0), comparing respective cohorts (CBD|800mg, CBD|600mg, CBD|800mg + Δ^9-THC|20mg, Δ^9-THC|10mg, placebo) to the average of all participants ("combined"). Asterix (*) denotes a significant variation in PS or t=0 measures (2way ANOVA, Benjamini, Krieger and Yekutiel corrected, $p<0.05$) for a particular cohort, compared to the combined average of that time-point measured.
