# Supplementary material for: Dose-dependent effects of oral cannabidiol and delta-9-tetrahydrocannabinol on serum anandamide and related N-acylethanolamines in healthy volunteers
Source: BMJ Ment Health. 2024 Aug 24;27(1):e301027. doi: 10.1136/bmjment-2024-301027 (PMC11409355; doi:10.1136/bmjment-2024-301027)
Supplement: online supplemental figure 2 [file bmjment-27-1-s002.pdf]

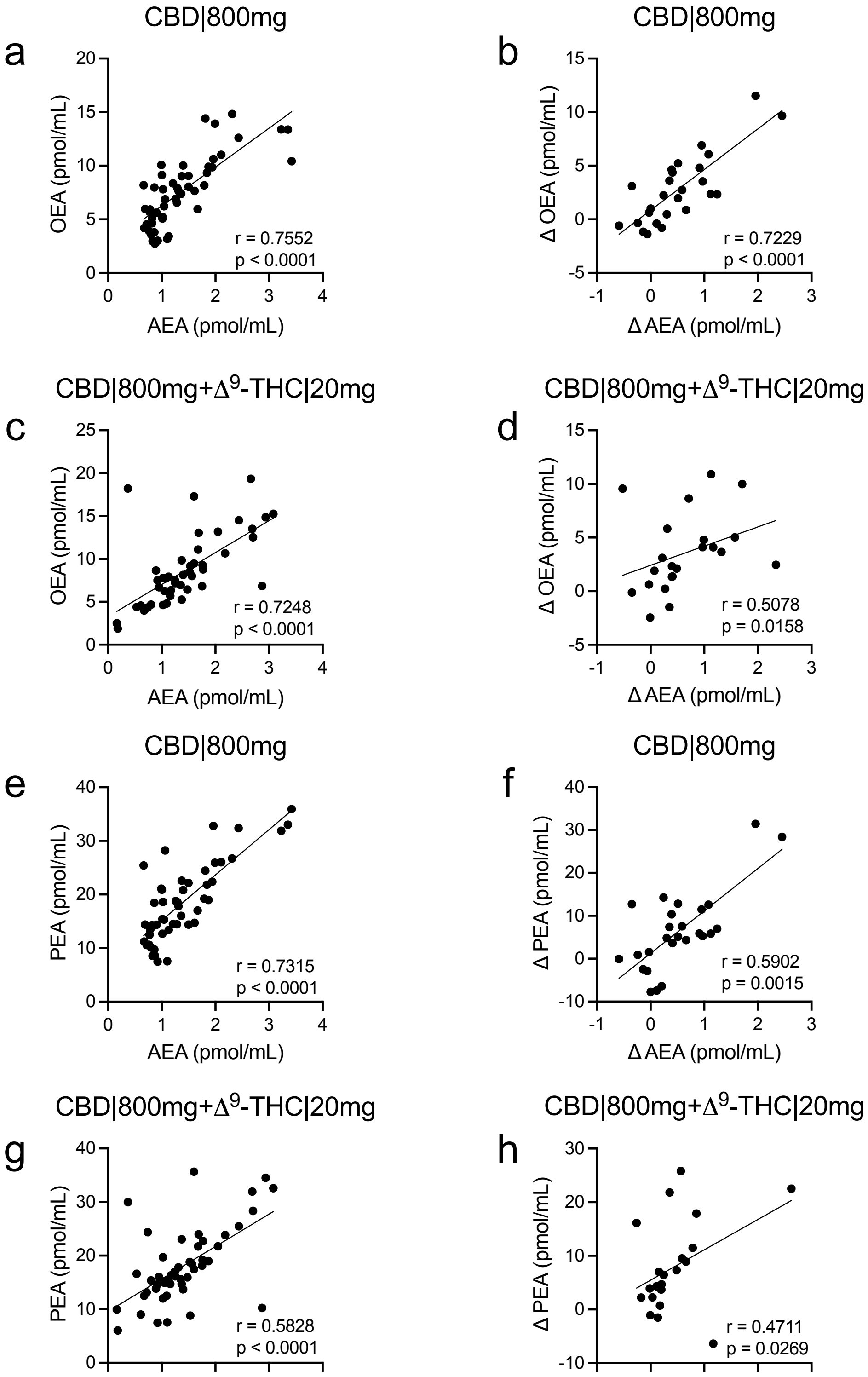

Supplementary Figure 2. Associations between AEA with the NAEs (A-D) OEA and (E-H) PEA for (A,B,E,F) CBD|800mg and (C,D,G,H) CBD|800mg + Δ^9-THC|20mg treatment arms. Correlations, determined by Spearman analysis at a confidence interval of 95%, were performed either as (A,C,E,G) a concentration comparison of OEA/PEA (y-axis, pmol/mL) to AEA (x-axis, pmol/mL) with all time-points included (PS, t=0, 65min and 165min post-intake); or (B,D,F,H) as differences at 65 and 165min from baseline (t=0) for OEA/PEA on the y-axis (Δ pmol/mL) compared to changes in AEA concentrations on the x-axis (Δ pmol/mL). The coefficient of correlation (r) and p-values are shown. List of abbreviations: CBD, cannabidiol; AEA, anandamide; OEA, oleoylethanolamide; PEA, palmitoylethanolamide.
