# Supplementary material for: Dose-dependent effects of oral cannabidiol and delta-9-tetrahydrocannabinol on serum anandamide and related N-acylethanolamines in healthy volunteers
Source: BMJ Ment Health. 2024 Aug 24;27(1):e301027. doi: 10.1136/bmjment-2024-301027 (PMC11409355; doi:10.1136/bmjment-2024-301027)
Supplement: online supplemental figure 3 [file bmjment-27-1-s003.pdf]

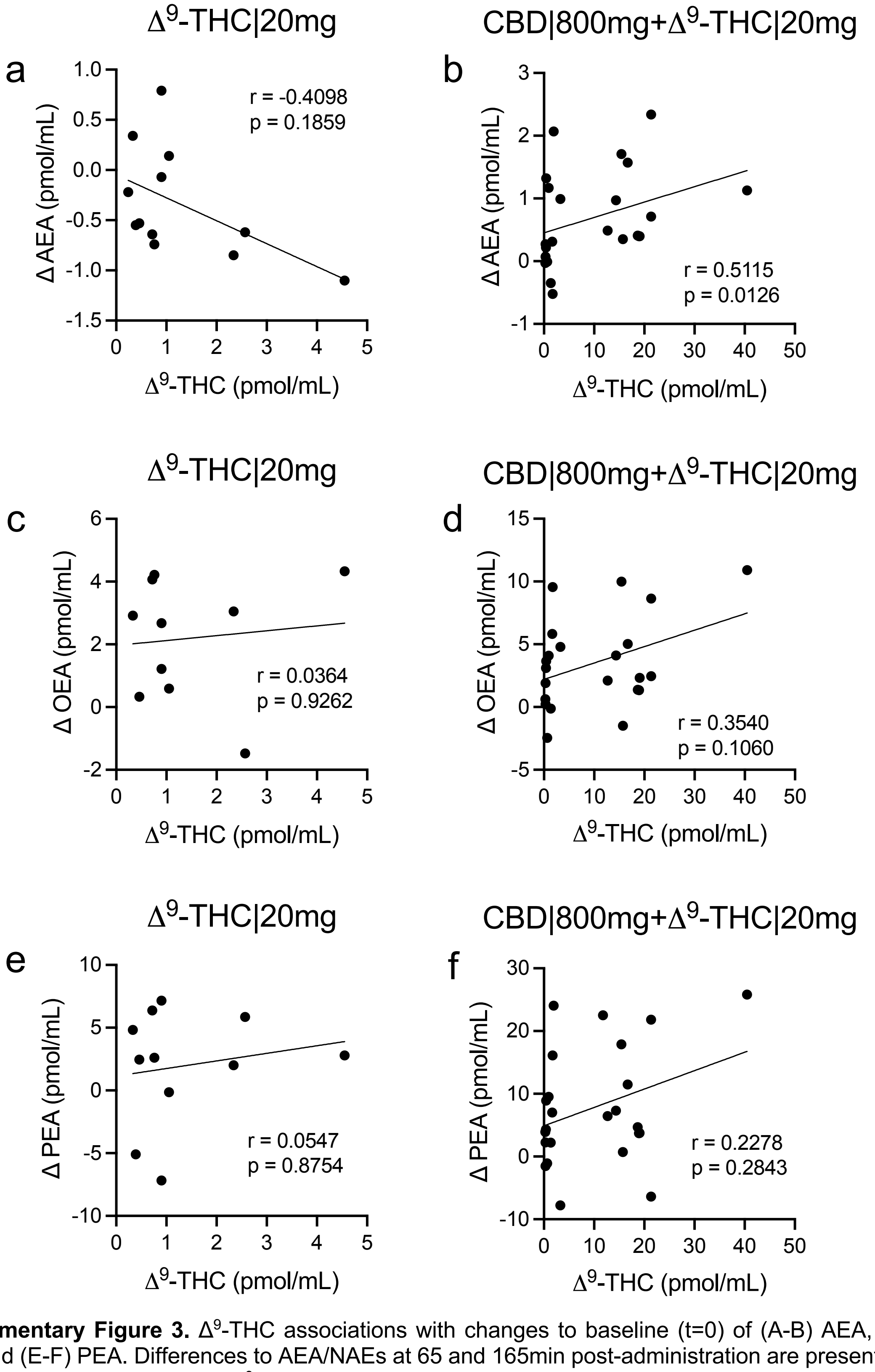

Supplementary Figure 3. Δ^9-THC associations with changes to baseline (t=0) of (A-B) AEA, (C-D) OEA and (E-F) PEA. Differences to AEA/NAEs at 65 and 165min post-administration are presented on the y-axis (Δ pmol/mL) against Δ^9-THC concentrations for same timepoints on the x-axis (pmol/mL) for (A, C, E) Δ^9-THC|20mg and (B, D, F) CBD|800mg + Δ^9-THC|20mg. Correlations were determined by Spearman analysis at a confidence interval of 95%. The coefficient of correlation (r) and p-values are shown. List of abbreviations: CBD, cannabidiol; AEA, anandamide; OEA, oleoylethanolamide; PEA, palmitoylethanolamide.
