# Supplementary material for: Dose-dependent effects of oral cannabidiol and delta-9-tetrahydrocannabinol on serum anandamide and related N-acylethanolamines in healthy volunteers
Source: BMJ Ment Health. 2024 Aug 24;27(1):e301027. doi: 10.1136/bmjment-2024-301027 (PMC11409355; doi:10.1136/bmjment-2024-301027)
Supplement: online supplemental table 1 [file bmjment-27-1-s004.pdf]

Supplementary Table 1. Operational parameters for TSQ Altis analysis of eCBs/NAEs.

Analyte	Precursor → Product (*m/z*)	CE [V]	RF Lens [V]
AEA	348.2 → 287.2<br>*348.2 → 62.2*	14.00<br>*18.00*	84<br>*84*
[$^2H_4$]-AEA	352.4 → 287.2<br>*352.4 → 66.2*	19.00<br>*14.00*	74<br>*74*
OEA	326.3 → 62<br>*326.3→ 44.1*	17.22<br>*41.94*	67<br>*67*
[$^2H_4$]-OEA	328.2 → 62<br>*328.2 → 44.1*	17.76<br>*45.35*	65<br>*65*
PEA	300.2 → 62<br>*300.2 → 44.1*	15.99<br>*18.94*	62<br>*62*
[$^2H_4$]-PEA	304.2 → 62<br>*304.2 → 44.1*	16.33<br>*19.79*	62<br>*62*
2-AG	379.3 → 287.2<br>*379.3 → 269.2*	19.00<br>*20.00*	89<br>*89*
[$^2H_4$]-2-AG	384.3 → 287.2<br>*384.3 → 269.2*	16.00<br>*17.00*	80<br>*80*
CBD/Δ^9-THC	315.2 → 193.2<br>*315.2 → 259.1*	22.31<br>*19.74*	74<br>*70*
[$^2H_4$]- CBD/[$^2H_4$]-Δ^9-THC	318.3 → 196.2<br>*318.3 → 262.1*	24.33<br>*19.79*	66<br>*66*

Transitions in *italics* were used as qualifier ions. List of abbreviations: CBD, cannabidiol; Δ^9-THC, delta-9-tetrahydrocannabinol; AEA, anandamide; 2-AG, 2-arachidonoylglycerol; OEA, oleoylethanolamine; PEA, palmitoylethanolamine; CE, collision energy; RF, Radio frequency; *m/z*, mass to charge ratio.
