# Supplementary material for: Dose-dependent effects of oral cannabidiol and delta-9-tetrahydrocannabinol on serum anandamide and related N-acylethanolamines in healthy volunteers
Source: BMJ Ment Health. 2024 Aug 24;27(1):e301027. doi: 10.1136/bmjment-2024-301027 (PMC11409355; doi:10.1136/bmjment-2024-301027)
Supplement: online supplemental table 2 [file bmjment-27-1-s005.pdf]

Supplementary Table 2. Mean values for CBD, Δ^9-THC eCBs and NAEs, per treatment group (pmol/mL), alongside individual and adjusted p-values representing changes in concentration (65 and 160min) from t=0

		Serum levels of eCBs/NAEs (pmol/mL)[1]			2-way ANOVA (comparison to t=0)			
		Post drug administration			65-min		160-min	
eCB/NAE	Medication	0min	65min	160min	p-value[2]	p_{corr}[3]	p-value[2]	p_{corr}[3]
AEA	Placebo	1.866 ± 0.200	1.559 ± 0.132	1.850 ± 0.154	0.0840	0.1765	0.9354	0.9822
	THC|10mg	2.554 ± 0.237	1.831 ± 0.130	2.182 ± 0.156	0.0013	***0.0014***	0.1670	0.0877
	THC|20mg	1.370 ± 0.098	1.031 ± 0.100	1.476 ± 0.113	0.0552	0.1160	0.5516	0.5791
	CBD|600mg	1.992 ± 0.198	1.703 ± 0.209	1.828 ± 0.175	0.1162	0.1534	0.1461	0.1534
	CBD|800mg	1.201 ± 0.149	1.530 ± 0.309	1.914 ± 0.302	0.0979	0.0514	0.0029	***0.0030***
	CBD|800mg + THC|20mg	1.169 ± 0.149	1.628 ± 0.268	2.480 ± 0.421	0.0624	***0.0328***	0.0076	***0.0080***
2-AG	Placebo	3.003 ± 0.461	3.046 ± 0.507	3.256 ± 0.832	0.8938	0.9385	0.6970	0.9385
	THC|10mg	3.448 ± 0.437	3.951 ± 0.758	4.746 ± 1.130	0.2396	0.2516	0.1154	0.2423
	THC|20mg	2.790 ± 0.326	2.919 ± 0.498	2.435 ± 0.366	0.8147	0.8554	0.4193	0.8554
	CBD|600mg	3.887 ± 0.683	3.305 ± 0.516	3.872 ± 0.824	0.0331	0.0694	0.9772	0.9999
	CBD|800mg	2.066 ± 0.338	2.717 ± 0.448	2.876 ± 0.664	0.1064	0.1773	0.1689	0.1773
	CBD|800mg + THC|20mg	2.633 ± 0.412	2.654 ± 0.401	3.020 ± 0.486	0.9421	0.9892	0.0483	0.1015
OEA	Placebo	9.322 ± 0.601	8.463 ± 0.669	10.088 ± 0.805	0.1496	0.2557	0.2435	0.2557
	THC|10mg	7.857 ± 1.002	6.296 ± 0.572	8.162 ± 0.773	0.2200	0.4621	0.6554	0.6882
	THC|20mg	7.857 ± 1.319	10.049 ± 0.783	9.758 ± 0.950	0.7295	0.9346	0.8901	0.9346
	CBD|600mg	6.894 ± 0.704	6.065 ± 0.567	6.714 ± 0.734	0.0995	0.2088	0.8536	0.8962
	CBD|800mg	6.692 ± 0.885	9.458 ± 1.480	9.577 ± 1.347	0.0126	***0.0132***	0.0072	***0.0132***
	CBD|800mg + THC|20mg	7.200 ± 0.870	12.225 ± 2.476	13.594 ± 2.549	0.0577	***0.0303***	0.0223	***0.0234***
PEA	Placebo	14.047 ± 1.703	15.586 ± 1.435	16.638 ± 1.871	0.2958	0.3106	0.1373	0.2883
	THC|10mg	20.320 ± 3.435	15.923 ± 1.892	21.190 ± 1.737	0.1152	0.2419	0.8200	0.8610
	THC|20mg	22.929 ± 2.065	23.294 ± 1.813	21.583 ± 2.013	0.8612	0.9042	0.5529	0.9042
	CBD|600mg	17.000 ± 1.713	19.622 ± 4.120	17.630 ± 1.740	0.5708	0.7796	0.7425	0.7796
	CBD|800mg	16.443 ± 2.063	22.907 ± 3.912	22.784 ± 3.456	0.0455	***0.0478***	0.0193	***0.0405***
	CBD|800mg + THC|20mg	16.294 ± 1.255	24.455 ± 3.912	28.775 ± 4.679	0.0991	0.0520	0.0181	***0.0190***

[1]Concentrations were expressed as mean ± standard error (SEM); [2]Unadjusted and [3]adjusted p-values for multiple comparisons using the two-stage Benjamini, Krieger and Yekutieli FDR method (p_{corr}) are reported for changes in eCB and NAE expression (65 and 160min) from baseline (0min), with significant values highlighted ($p < 0.05$, underlined) and emphasised when adjusted for FDR ($p_{corr} < 0.05$, ***bold italics underlined***). Abbreviations, eCB: endocannabinoid; NAE: N-acylethanolamine; HVs: healthy volunteers; AEA: anandamide; 2-AG: 2-arachidonoylglycerol; OEA: oleolylethanolamine; PEA: palmitoylethanolamine.
